# Mechanistic Insights into Radiation Resistance in Colorectal Cancer: Gene Exploration Study

**DOI:** 10.3390/ijms26083849

**Published:** 2025-04-18

**Authors:** Beamjun Park, Soohyeon Lee, Inyoung Jo, Donghyun Kang, Taewan Kim, Jaesung Ryu, Hyejeong Kong, Moojun Baek, Taesung Ahn

**Affiliations:** 1 Department of Medical Life Science, Soonchunhyang University, Asan 31538, Republic of Korea; cool373867@gmail.com (B.P.); ktwdreem@gmail.com (T.K.); rjs950826@gmail.com (J.R.); angelkonghj@gmail.com (H.K.); 2 Department of Colorectal Surgery, College of Medicine, Soonchunhyang University Cheonan Hospital, Cheonan 31151, Republic of Korea; marchsh93@gmail.com (S.L.); c100048@schmc.ac.kr (D.K.);; 3 Department of Radiation Oncology, Soonchunhyang University, Asan 31538, Republic of Korea; iyoung.jo@schmc.ac.kr

**Keywords:** colorectal cancer cell, radiation resistance, key genes in radiation resistance

## Abstract

Radiotherapy is a cornerstone of colorectal cancer (CRC) treatment; however, its therapeutic efficacy is often compromised by both intrinsic and acquired resistance in CRC cells. This study employed small interfering RNA (siRNA) technology to elucidate the functional roles of BAMBI, GADD34, NFKBIA, and NFKBID in CRC cell lines SW480 and HCT116. We assessed their impact on key cellular processes and radiation sensitivity. Gene silencing of all four target genes significantly suppressed CRC cell proliferation, migration, and invasion. Moreover, siRNA-mediated knockdown enhanced radiation sensitivity, as evidenced by a substantial increase in apoptosis and a marked reduction in cell viability compared with controls. These findings suggest that BAMBI, GADD34, NFKBIA, and NFKBID serve as critical regulators of CRC progression and radiation resistance. Overall, this study provides a mechanistic foundation for further exploration into the pathways underlying radiation resistance and underscores the potential for developing personalized radiotherapy strategies guided by molecular profiling.

## 1. Introduction

Colorectal cancer (CRC) has shown a decrease in incidence over the past 10 years; however, it remains the second most common cause of cancer-related death, following lung cancer [1,2,3,4]. Recent studies have explored approaches to overcoming radioresistance from an epigenetic perspective, identifying regulatory mechanisms and gene targets involved in this phenomenon [5,6]. In several cancer types, clinical trials have been initiated to target specific genes associated with key signaling pathways known to contribute to radioresistance, including the ERK/MAPK, Wnt/β-catenin, TGF-β/SMAD, and PI3K-AKT pathways. In rectal cancer, radiation resistance has been found to be associated with chromosomal alterations on 1p, elevated carcinoembryonic antigen levels that stimulate M2 macrophage polarization, and miR-31-regulated DNA repair genes. However, no clinically validated or reliable biomarkers have yet been established for predicting or guiding radiotherapy responses [7].

In the present study, we analyzed tissue samples collected from colorectal cancer patients to identify genes that are differentially expressed between normal and tumor tissues, as well as before and after radiotherapy. From this analysis, four genes—BAMBI, GADD34, NFKBIA, and NFKBID—were selected for further investigation. Using siRNA-mediated gene silencing and a series of functional assays, we aimed to determine whether modulation of these genes affects CRC cell behavior and radiosensitivity.

## 2. Results

### 2.1. CRC Patient Data

**Figure 1:**
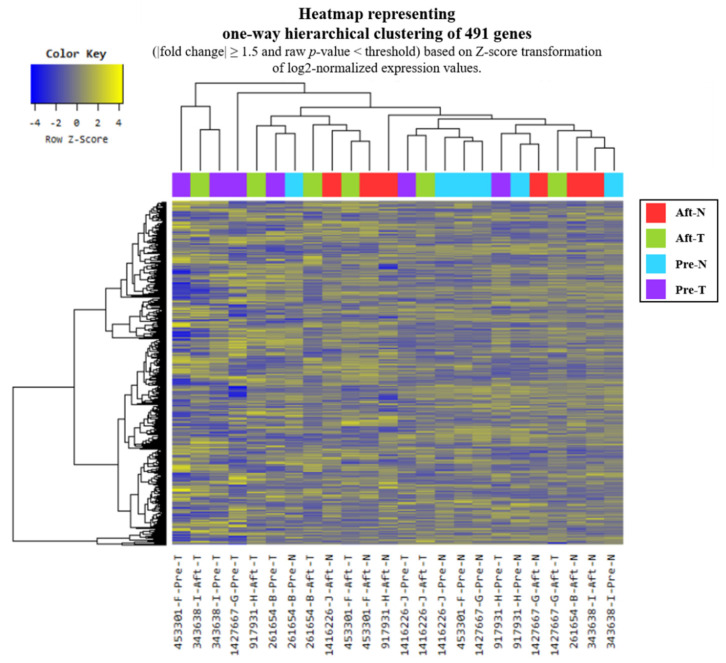
Heatmap for DEG list. A diagram visualizing the information grouped by sample group and by gene using the degree of similarity of expression patterns for each gene through hierarchical clustering (distance metric = Euclidean distance, linkage method = complete) analysis for significant genes. (Aft-N; after radiotherapy—normal tissue, Aft-T; after radiotherapy—tumor tissue, Pre-N; pre-radiotherapy—normal tissue, Pre-T; pre-radiotherapy—tumor tissue).

**Figure 2:**
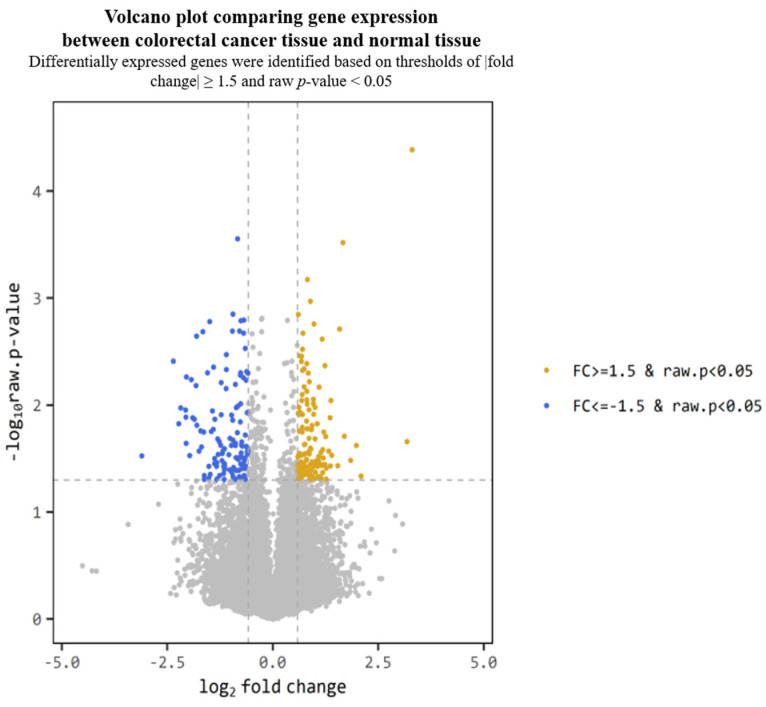
Volcano plot showing differentially expressed genes between tumor and normal tissues. Genes with fold change ≥ 1.5 and *p* < 0.05 are highlighted. (*X*-axis: log2 fold change, *Y*-axis: −log10 *p*-value).

NGS was performed on normal and tumor tissues collected from six CRC patients, before and after radiotherapy. A total of 491 significant genes were identified based on a fold change ≥ 1.5 and a raw *p*-value < 0.05 (Figure 1). Among these, genes that were distinctly expressed in tumor tissues compared with normal tissues were visualized using a volcano plot (Figure 2). Of the 39 candidate genes that showed significant expression changes in tumor tissues before and after radiotherapy, four genes—BAMBI, GADD34, NFKBIA, and NFKBID—were finally selected based on fold change values, prior literature, and relevance to CRC as reported in the Human Protein Atlas and previous studies.

### 2.2. Cell Function Tests After Gene Knockdown in the CRC Cell Line

**Figure 3:**
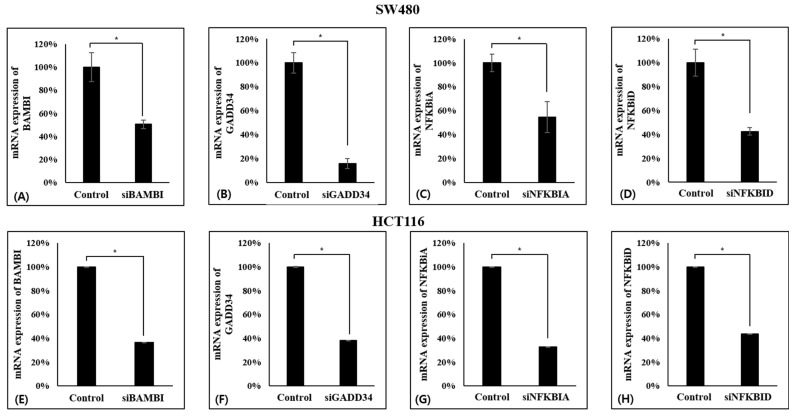
Gene knockdown verification in the CRC cell line using real-time PCR. Expression of siBAMBI (**A**,**E**), siGADD34 (**B**,**F**), siNFKBIA (**C**,**G**), siNFKBID (**D**,**H**) genes in SW480 and HCT116. * *p* < 0.01.

The expression levels of BAMBI, GADD34, NFKBIA, and NFKBID were confirmed at the mRNA level in CRC cell lines SW480 and HCT116. It was necessary to confirm the effect of gene expression on cell function. SW480 and HCT116 cell lines with knockdown of the four genes via siRNA were established, and it was confirmed that the expression levels of the four genes were reduced by more than 60% in all CRC lines (Figure 3).

**Figure 4:**
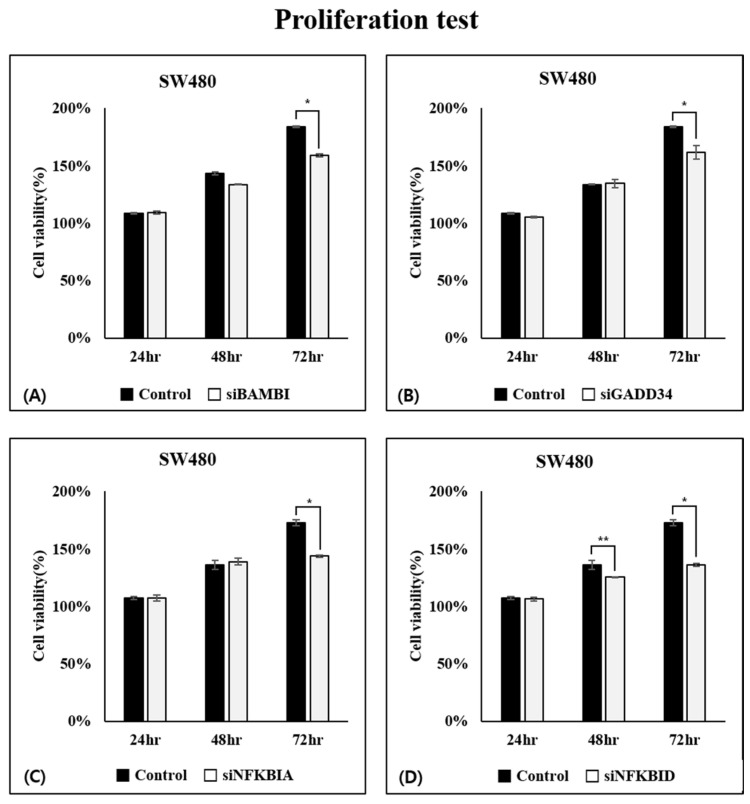
Cell proliferation test after gene knockdown in the CRC cell line. siBAMBI (**A**), siGADD34 (**B**), siNFKBIA (**C**), siNFKBID (**D**). * *p* < 0.01, ** *p* < 0.05.

We compared untreated CRC cell lines (control group) with cell lines in which each of the four genes underwent individual knockdown. First, a cell proliferation test was conducted. During the initial 48 h, no significant differences were observed in the BAMBI, GADD34, and NFKBIA knockdown cell lines. However, the NFKBID knockdown cell line exhibited reduced cell viability compared with the control. After 72 h, cell proliferation was found to have decreased by more than 20% in all gene knockdown groups, which was statistically significant. These results reveal that the knockdown of the four genes negatively regulated the proliferation of CRC cells (Figure 4).

**Figure 5:**
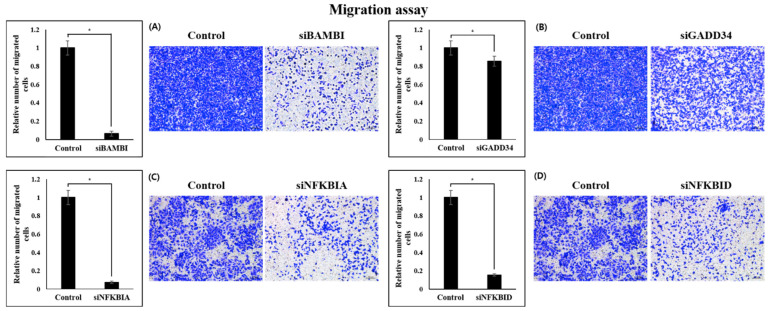
Migration assay results of the control and gene knockdown groups in SW480 cells. After crystal violet staining, migrated cells were visualized using a light microscope and quantified by manual counting in at least three randomly selected fields per condition. * *p* < 0.01. siBAMBI (**A**), siGADD34 (**B**), siNFKBIA (**C**), siNFKBID (**D**).

Migration and invasion of colon cancer cells were investigated according to the expression of four genes. It was confirmed that the ability of all CRC cell lines was significantly reduced when four genes were knocked down. In migration, there was found to be a significant difference of the knockdown cell line of four genes. The BAMBI knockdown cell lines confirmed a nine-times lower cell migration ability compared with the control group, and both NFKBIA and NFKBID showed reduced ability by more than 80% (Figure 5). Cell line knockdown of the GADD34 gene showed significant differences in migration ability, but did not show high differences compared with the other three genes.

**Figure 6:**
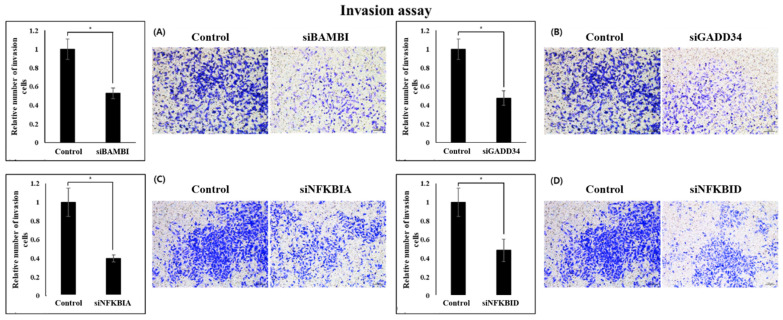
Invasion assay results of the control and gene knockdown groups in SW480 cells. After crystal violet staining, invaded cells were visualized using a light microscope and quantified by manual counting in at least three randomly selected fields per condition. * *p* < 0.01. siBAMBI (**A**), siGADD34 (**B**), siNFKBIA (**C**), siNFKBID (**D**).

When the expression of BAMBI, GADD34, NFKBIA and NFKBID genes was downregulated, it was confirmed that the invasion ability was significantly reduced. In all knockdown cell lines, metastatic capacity decreased by more than 50%, indicating that all four genes are involved in the function of colon cancer cells (Figure 6). All these data indicate that BAMBI, GADD34, NFKBIA and NFKBID genes the enhance proliferation, migration, and invasion of CRC cells.

### 2.3. Gene Expression in the CRC Cell Line to Radiation Sensitivity and Sensibility

**Figure 7:**
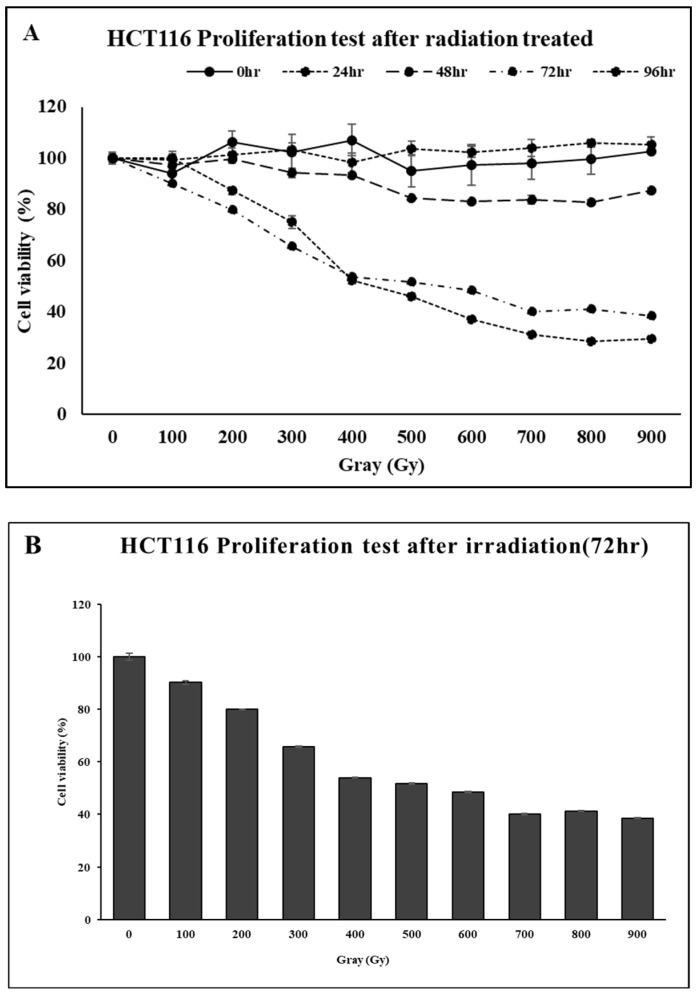
Assessment of radiation sensitivity in CRC cell lines based on cell viability. (**A**) Time-dependent changes in cell viability following irradiation. (**B**) Dose-dependent differences in cell viability assessed 72 h after irradiation.

To determine the appropriate radiation dose and incubation time for assessing radiosensitivity in CRC cell lines, cell viability was measured following irradiation. Ionizing radiation was applied at doses ranging from 0 to 900 Gy in 100 Gy increments, and cell viability was assessed at 0, 24, 48, 72, and 96 h post-irradiation (Figure 7A). A reduction of more than 50% in cell viability was observed at 72 and 96 h. To determine the half-maximal inhibitory concentration (IC_50_), the incubation time was fixed at 72 h, and cell viability was re-evaluated across the same radiation dose range. As shown in Figure 7B, a dose of 500 Gy reduced cell viability to approximately 50%. Based on these results, subsequent experiments evaluating radiosensitivity following gene knockdown were conducted under the condition of 500 Gy irradiation with a 72-h incubation period.

**Figure 8:**
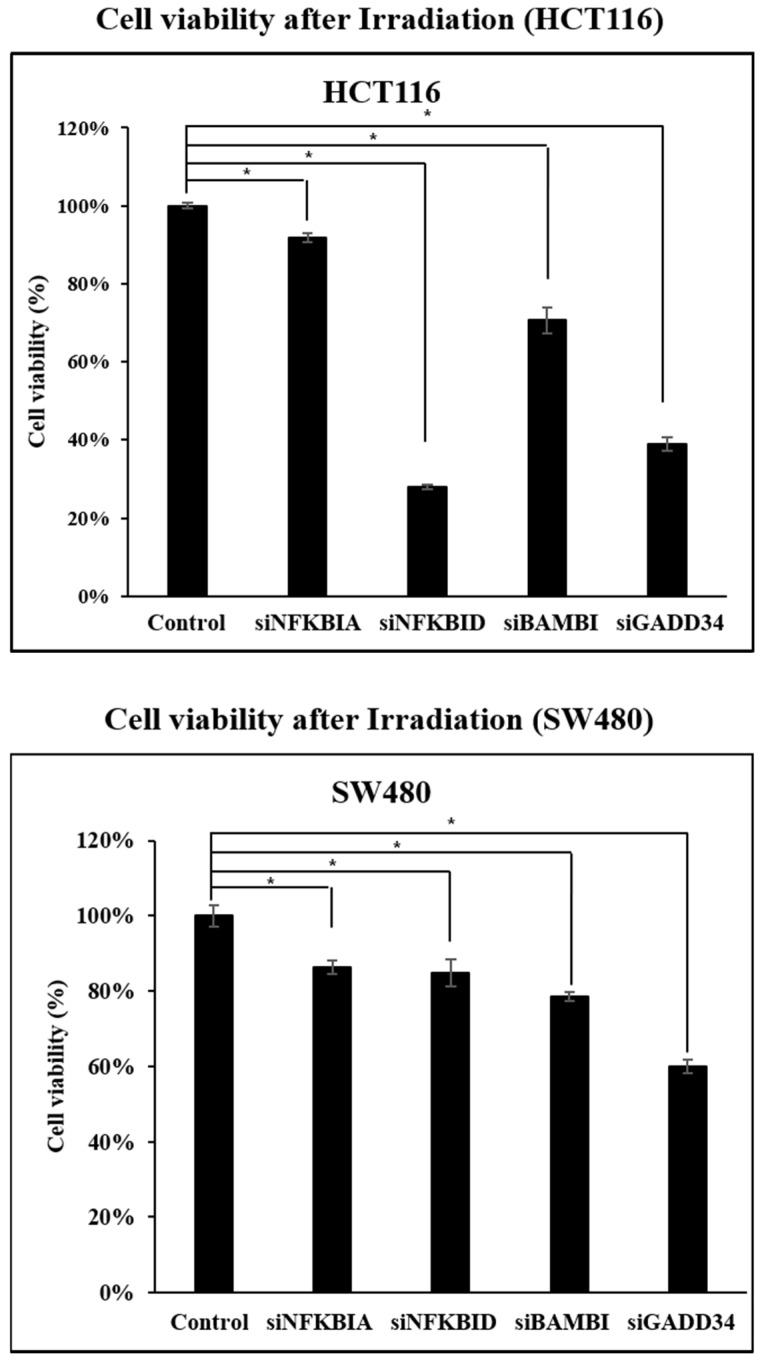
Assessment of cell viability following gene knockdown to evaluate radiosensitivity. * *p* < 0.01.

HCT116 and SW480 cell lines were subjected to 500 Gy of ionizing radiation, and cell viability was evaluated 72 h post-irradiation. A comparative analysis was conducted between control cells and those with individual knockdown of the four target genes. Cell viability was quantified based on absorbance measurements using the Ez-Cytox reagent. When the viability of control cells was defined as 100%, all gene knockdown groups exhibited a statistically significant reduction in post-radiation survival. Among the four genes, NFKBIA showed the smallest reduction, whereas NFKBID demonstrated cell-line-dependent differences in viability. Notably, knockdown of BAMBI and GADD34 resulted in a pronounced decrease in survival, with cell viability falling below 80% in both cell lines (Figure 8).

### 2.4. Confocal Image of Radiation Sensitivity According to Gene Expression

**Figure 9:**
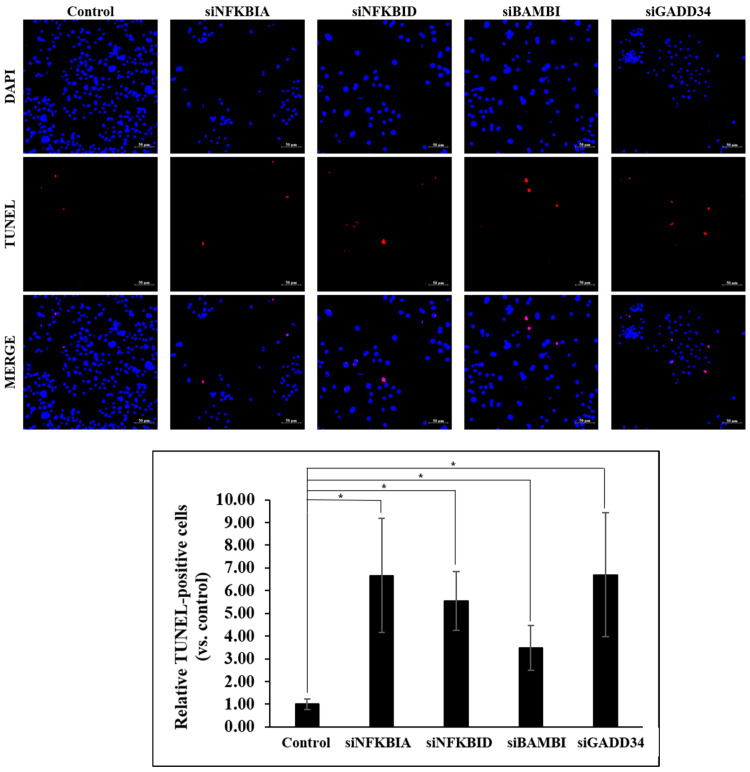
Cell death rate following irradiation. Representative confocal images of DAPI and TUNEL staining, along with quantitative analysis comparing the proportion of TUNEL-positive cells among total cells in control and gene knockdown groups. * *p* < 0.01.

To visually evaluate cell survival following irradiation, both control and gene knockdown groups were processed using the same protocol as described in the Ez-Cytox viability assay and subsequently imaged using confocal microscopy. Cells were seeded at equal densities on coverslips, irradiated with 500 Gy, and incubated for 72 h. Nuclei were stained with DAPI (blue fluorescence) to visualize total cell numbers. The number of DAPI-positive nuclei was notably higher in the control group compared with the four gene knockdown groups, indicating a significant reduction in proliferating cells upon gene silencing. To assess the extent of cell death, apoptotic cells were stained using TUNEL reagents (red fluorescence), and both total and TUNEL-positive cells were manually counted in four randomly selected microscopic fields per group. The proportion of TUNEL-positive cells relative to the total cells was calculated, and the mean values with standard deviations were determined. All gene knockdown groups exhibited at least a threefold increase in the number of apoptotic (TUNEL-positive) cells compared with the control group, indicating enhanced radiosensitivity upon gene silencing (Figure 9).

## 3. Discussion

This study addresses a critical gap in understanding colorectal cancer (CRC) radiation resistance by identifying BAMBI, GADD34, NFKBIA, and NFKBID as key modulators of tumor progression and response to radiotherapy. These findings offer new insights into the molecular mechanisms driving resistance and suggest potential therapeutic targets to enhance treatment outcomes.

Our findings demonstrate that silencing these genes significantly suppresses CRC cell proliferation, migration, and invasion while enhancing radiation-induced apoptosis. Although the extent of these effects varied among individual genes, proliferation and invasion assays consistently showed comparable reductions across all experimental groups relative to the control. However, in the cell migration assay, knockdown of BAMBI, NFKBIA, and NFKBID resulted in a substantial decrease of over 80%, whereas the reduction observed for GADD34 was comparatively modest.

These findings suggest that the observed effects may be attributed to GADD34-mediated upregulation of myosin IIA expression within the colonic intestinal epithelium, which serves to inhibit cell migration [8,9]. Conversely, the significant reduction in proliferation and invasion is likely associated with STAT3 signaling. GADD34 has been shown to enhance the production of pro-inflammatory mediators, including TNF-α and IL-6, which subsequently activate STAT3 signaling, thereby facilitating colonic epithelial cell proliferation [10]. The IL-6/JAK/STAT3 signaling pathway plays a critical role in regulating the tumor microenvironment, thereby promoting tumor growth, invasion, and metastasis. In addition, STAT3 has been implicated in DNA damage repair. Studies have shown that knockdown of STAT3 impairs DNA repair efficiency by downregulating the ATM–Chk2 and ATR–Chk1 pathways [11]. GADD34 has been shown to contribute to the activation of STAT3 signaling, suggesting that it may influence radiosensitivity through this regulatory mechanism.

In our study, the NF-κB inhibitor, NFKBIA (IκB-α), functions by maintaining NF-κB in an inactive state, thus suppressing its activity. On the other hand, NFKBID is a component of the Th17/Treg NF-κB signaling cascade and is essential for Th17 differentiation in response to inflammation and infection, although it does not appear to influence apoptosis. NF-κB, one of the most extensively studied transcription factors, operates through both canonical and non-canonical signaling pathways. It is activated in response to various stressors, including irradiation, and plays a pivotal role in promoting cell survival and proliferation by interacting with crucial pathways such as PI3K-AKT-mTOR and STING. Radiation has been shown to activate the DNA-binding activity of NF-κB. Inhibition of this activation leads to increased cell death and reduced cell proliferation and clonogenic survival in certain cancer types. Moreover, suppression of NF-κB has been reported to block the development of adaptive radioresistance. However, such responses are not universally observed across all cancer types.

Our findings demonstrate that silencing NFKBIA and NFKBID results in a reduction of CRC cell viability following radiotherapy, suggesting that NF-κB activation may enhance the therapeutic efficacy of radiation. However, contrary studies have posited that inhibition of NF-κB activation promotes apoptosis, thereby improving the effectiveness of radiotherapy (RT) and chemotherapy (CT) [12]. The effects of NF-κB modulation appear to be context-dependent, with pathway-specific outcomes. Inhibition of the canonical NF-κB pathway has been shown to suppress irradiation-induced antitumor immune responses, thereby diminishing RT efficacy, while inhibition of the non-canonical pathway can regulate type I interferon (IFN) expression, thereby enhancing antitumor immunity via dendritic cells and CD8+ T cells [13,14]. Given the rapid and transient nature of canonical NF-κB activation, the timing of post-radiation cell viability assessments in our study may have primarily captured the early-phase response. Therefore, further investigation into the temporal dynamics of NF-κB signaling is warranted. Additionally, due to the biphasic nature of NF-κB signaling, significant challenges remain in targeting NF-κB as a therapeutic strategy for CRC treatment.

BAMBI, a TGF-β pseudoreceptor, functions as a negative regulator of the TGF-β/SMAD signaling pathway. While its role in cancer has been primarily investigated in the context of metastatic disease, its broader implications remain less well understood. Notably, TGF-β signaling has been shown to suppress migration and metastasis in CRC, whereas, in other malignancies, it facilitates epithelial–mesenchymal transition and enhances metastatic potential [15]. Previous studies have reported an absence of BAMBI overexpression in non-metastatic CRC. However, our findings indicate that, even in CRC without distant metastases, BAMBI exerts a significant influence on cell proliferation, migration, and invasion. These results align with prior studies demonstrating that BAMBI knockdown reduces the expression of Wnt10B, p53, and Bcl-2, leading to a concomitant decrease in cell proliferation [16]. TGF-β, one of the most well-known mediators associated with radioresistance, has been shown to promote resistance through the induction of EMT, cancer stem cells, and cancer-associated fibroblasts. Notably, dysregulation of TGF-β signaling has been observed in various tumor types that exhibit radioresistance [4]. To explore this relationship, Wang et al. utilized murine MC38 colon cancer and B16 melanoma cell lines, demonstrating that radiation-mediated downregulation of BAMBI in myeloid-derived suppressor cells (MDSCs) enhances TGF-β signaling, thereby contributing to extrinsic radiation resistance [17]. Based on these findings, the authors proposed that BAMBI overexpression during radiotherapy could improve local tumor control by counteracting extrinsic radiation resistance. However, their study did not assess the role of tumor-cell-intrinsic BAMBI, and its translational relevance remains limited due to the use of murine rather than human CRC cell lines. Moreover, TGF-β signaling exerts tumor-suppressive functions by regulating cytostasis, differentiation, and apoptosis. Notably, prior studies have reported that BAMBI overexpression in ovarian cancer promotes cell proliferation and migration, while also reducing TGF-β-induced apoptosis more than fourfold [18,19]. In our study, the knockdown of BAMBI prior to radiotherapy resulted in a notable reduction in cell viability, indicating that the expected inhibition of TGF-β by BAMBI was not occurring. This likely facilitated an increase in TGF-β-mediated apoptosis compared with the control group. Therefore, BAMBI, a known regulator of the TGF-β/Smad signaling pathway, is thought to modulate radiosensitivity through its influence on this mechanism. It is essential to investigate the temporal dynamics and mechanistic alterations of TGF-β signaling following the initiation of radiotherapy, with particular attention to changes in BAMBI expression and activity. These insights could inform the strategic modulation of BAMBI expression to enhance therapeutic efficacy.

Our study identifies these genes as critical regulators of CRC progression and radiotherapy response, underscoring the need for personalized therapeutic strategies that modulate gene expression and present novel therapeutic targets. However, several limitations must be considered. As this study was conducted in vitro, further validation is necessary to ascertain whether these findings translate to clinical settings. Consequently, follow-up investigations employing animal models and clinical samples are imperative. Moreover, the mechanistic interactions between these genes and tumor immune responses remain insufficiently characterized. CRISPR-Cas9-based gene editing and rescue experiments were not performed in the current study. Future investigations will aim to validate gene-specific effects through these approaches to further elucidate their mechanistic relevance.

## 4. Materials and Methods

### 4.1. Gene Selection

Tissue samples were obtained from six patients newly diagnosed with colorectal cancer. Paired normal and tumor tissues were collected via sigmoidoscopic biopsy prior to radiotherapy and again four weeks after the completion of radiotherapy. Next-generation sequencing (NGS) analysis was subsequently conducted to identify differentially expressed genes associated with the radiotherapy response. Genes with a fold change ≥ 1.5 and a raw *p*-value < 0.05 were considered statistically significant.

### 4.2. Cell Lines and Culture

Among various colorectal cancer cell lines, HCT116 and SW480 were selected for this study based on their consistent proliferation rates, stable culture conditions, and high siRNA transfection efficiency, all of which contribute to reliable experimental reproducibility. HCT116 is characterized by its rapid growth rate, while SW480 exhibits low metastatic potential and relatively low radiosensitivity. Both cell lines were obtained from the Korea Cell Line Bank (Korean cell line bank, Seoul, Republic of Korea). Cells were grown in RPMI 1640 medium supplemented with 10% fetal bovine serum (FBS) and 1% penicillin streptomycin solution (ABS) at 37 °C in a humid atmosphere containing 5% CO_2_.

### 4.3. Small Interfering RNA(siRNA) and Transient Transfection

Human CRC cell lines were seeded with 2 × 10^6^ cells in 10cm cell culture dish. After 24 h, the medium was changed to siRNA, Opti MEM (Gibco, Massachusetts, USA, 31985-062) and Hiperfect^®^ (QIAGEN, Germany, 301707) were mixed and replaced the existing media. siRNA was used to silence genes. Preliminary experiments were performed using three siRNA concentrations: 0.5×, 1×, and 2× the recommended dose, in order to determine optimal transfection conditions. Based on these trials, the optimal knockdown efficiency was achieved at a concentration of 20 nmol/μL with a 48-h incubation period. Cell function tests and radiosensitivity assays were subsequently performed using cells transfected under these optimized conditions. To establish appropriate control conditions, an additional set of preliminary experiments was carried out by varying the cell seeding density (3 × 10^5^, 5 × 10^5^, and 7 × 10^5^ cells per well) and incubation times (24, 48, and 72 h). From these experiments, the optimal experimental parameters were determined. All main experiments were performed in triplicate, and average values were calculated.

### 4.4. Cell Proliferation

The cells were cultured in T-75 flasks and subcultured when reaching approximately 80–90% confluency. For subculturing, cells were seeded at a density of 1 × 10^6^ cells per flask. To perform the experiment, cells were seeded into 96-well plates containing membranes. At 24, 48, 72 h after incubation, Ez-Cytox solution was added to each well, followed by a 2-h incubation in a humidified incubator at 37 °C with 5% CO_2_. Absorbance was subsequently measured at 450 nm using a microplate reader (ThermoFisher, Waltham, MA, USA, 51119200).

### 4.5. Transwell Migration Assay

A migration assay was performed to evaluate the migratory capacity of the cells, using a 24-well plate equipped with a 6.5 mm polycarbonate membrane insert with a pore size of 8.0 μm (Corning, Somerville, MA, USA). A total of 250 μL of RPMI 1640 medium containing 3 × 10^5^ cells was added to the upper chamber. In the bottom chamber, 750 μL of RPMI 1640 medium, containing 10% FBS and 1% ABS, was added. This was incubated for 48 h in a humidified incubator containing CO_2_ and at 37 °C. Then, the medium in the bottom chamber and the Transwell chamber was removed and the chamber was washed three times using phosphate buffered saline (PBS). To fix the cells in the membrane of the washed chamber, 4% formaldehyde was applied to the Transwell and bottom chambers and reacted for 2 m. After washing 3 times with PBS, 100% methyl alcohol was applied and reacted for 10 m. In addition, cells were stained by immersing the Transwell chamber in 0.5% crystal violet solution for 2 m. Using an optical microscope, 5 sites of the membrane were randomly photographed (900 × 1200 µm^2^).

### 4.6. Transwell Invasion Assay

Invasion assays were performed using 24-well inserts with 6.5 mm polycarbonate membranes and a pore size of 8.0 μm (Corning, Somerville, MA, USA). The inside of the Transwell was covered with a non-growth factor 1:4 ratio Matrigel dilution in RPMI 1640 and incubated for 1 h at 37 °C. A concentration of 5 × 10^5^ cells was then plated in the insert cells and 750 μL of RPMI 1640 medium with 10% FBS and 1% ABS was added in the bottom chamber. After 72 h of incubation, cells were fixed with 4% formaldehyde and stained with 0.05‰ crystal violet.

### 4.7. Terminal Deoxynucleotidyl Transferase dUTP Nick End Labeling (TUNEL) Assay

Cells were seeded in the 12-well plate with an added cover slide. The TUNEL assay kit detects the DNA fragmentation of apoptotic cells by marking DNA breaks using standard immunohistochemical techniques. Thus, after treatment with the nanoparticles, the cells were fixed by incubation in 4% formaldehyde for 30 min. Cells were permeabilized using 0.2% Triton X-100 (in PBS) for 45 min at 37 °C to facilitate staining. All wells received a DNA marking solution (10 µL of reaction buffer, 0.75 µL of TdT enzyme, 8.0 µL of BrdUTP and 31.25 µL of dH_2_O) and were incubated for 60 min at 37 °C. The nuclei were counterstained with DAPI.

### 4.8. Radiation Irradiation

HCT116 colorectal cancer (CRC) cells were subjected to varying doses of radiation to evaluate their response to irradiation. Cells were irradiated with doses ranging from 0 to 900 Gray (Gy) in 100 Gy increments using a calibrated linear accelerator. Cell viability was measured at 0, 24, 48, 72, and 96 h post-irradiation to determine the impact of radiation dose and exposure time. Based on the survival data, 500 Gy was selected as the optimal dose for further experiments. Subsequent irradiation of HCT116 cells was conducted at 500 Gy to assess the effect of radiation on cell proliferation and viability under various experimental conditions. Cell survival was evaluated using the Ez-Cytox assay, and apoptotic cell populations were analyzed using TUNEL staining as described in the earlier sections.

### 4.9. Real-Time Polymerase Chain Reaction (PCR)

Colon cancer cells were seeded at a density of 1 × 10^4^ cells per well in 6-well cell culture plates. These were cultured after 24 h, and 1ml RiboEx (GeneAll, Seoul, Republic of Korea, 301-001) was added to each well according to the manufacturer’s protocol. Total RNA was isolated from all cell lines using the Hybrid-R™ kit (GeneAll, Seoul, Republic of Korea, 305-101). Reverse transcription was performed using ReverTra Ace™ kit (TOYOBO, Osaka, Japan, FSQ-101). Quantitative qRT-PCR was performed using the SYBR^®^ Green (TOYOBO, Osaka, Japan, QPK-201). The PCR cycle included one cycle at 95 °C for 1 m, followed by 39 cycles at 95 °C for 15 s, 60 °C at 15 s, and 72 °C for 25 s. The primers used for real time PCR were based on data from the ensemble (Table 1).

### 4.10. Statistical Analysis

In order to improve the reproducibility and statistical robustness of our experiments, each experiment was conducted at least three times. The graphs were constructed based on the mean values and standard deviations derived from these repeated measurements. Statistical analyses were performed using SPSS version 26.0 for Windows 11 (SPSS Inc., USA). Student’s *t*-tests were used to compare knockdown validation, cell proliferation, migration, and invasion assays in CRC cell lines. A *p*-value of <0.05 was considered statistically significant. Survival outcomes were analyzed using the Kaplan–Meier method. In graphical representations, *p* < 0.01 was indicated by a single asterisk (*), and *p* < 0.05 was indicated by a double asterisk (**).

## 5. Conclusions

Colorectal cancer (CRC) is characterized by significant tumor heterogeneity, leading to substantial variability in individual patient responses. Moreover, a complex array of mechanisms underlies both the early and late phases of response to radiotherapy. Through gene silencing experiments, we demonstrated that the knockdown of BAMBI, GADD34, NFKBIA, and NFKBID significantly suppresses CRC cell proliferation, migration, and invasion, while simultaneously enhancing radiation-induced apoptosis. In conclusion, this study provides a scientific foundation for enhancing radiotherapy efficacy and mitigating treatment resistance in CRC. Furthermore, these findings underscore the potential for developing targeted therapeutic strategies that could ultimately improve patient survival outcomes.

## Figures and Tables

**Table 1:** Primer of key genes of radiation resistance.

Gene ID	Primer		Primer Sequence (5’ –> 3’)	Length	Ensembl Genes
25805	BAMBI	Forward	GCTGCACGATGTTCTCCTCTC	20	ENSG00000095739
		Reverse	ATCTGTTGCCGCGTATCCTG	20	
4792	NFKBIA	Forward	ACGAGGAGTACGAGCAGATG	20	ENSG00000100906
		Reverse	TGGAAGTTGAGGAAGGCCAG	20	
84807	NFKBID	Forward	ATCAGGGACGTTCGGTCTTG	20	ENSG00000167604
		Reverse	TGGACACAATCCAGCCTGTC	20	
23645	GADD34	Forward	CCTAAAGGCCAGAAAGGTGC	20	ENSG00000087074
		Reverse	GGGCTAAAGGTGGGTTCCTG	20	

## Data Availability

Data will be made available on request.
